# Radiographic findings in dogs from an endemic area for heartworm disease in the state of Rio de Janeiro

**DOI:** 10.29374/2527-2179.bjvm008324

**Published:** 2025-01-02

**Authors:** Nathália da Conceição Lima, Mel Andrade Souza de Paula, Rebeca Bhering Mattos, Denise do Valle Soares, Giovanna Pestana Silva, Bruno Alberigi

**Affiliations:** 1 Veterinarian, Programa de Pós-graduação em Medicina Veterinária (PPMV), Departamento de Clínica e Cirurgia Veterinária (DMCV), Instituto de Veterinária (IV), Universidade Federal Rural do Rio de Janeiro (UFRRJ), Seropédica, RJ, Brazil.; 2 Undergraduate in Veterinary Medicine, IV, UFRRJ, Seropédica, RJ, Brazil.; 3 Veterinarian, Autonomous, Rio de Janeiro, RJ, Brazil; 4 Veterinarian, DSc, DMCV, IV, UFRRJ, Seropédica RJ, Brazil.

**Keywords:** radiography, pulmonary artery, parasitosis, image diagnosis, heartworm disease, radiografia, artéria pulmonar, parasitose, diagnóstico por imagem, dirofilariose. canina

## Abstract

Canine heartworm disease causes significant pulmonary abnormalities, even in asymptomatic cases. This study aimed to compare the chest radiographs of cases infected with *Dirofilaria immitis* with those of heartworm-negative dogs. Fifteen animals treated at a private veterinary clinic in the municipality of Maricá, Rio de Janeiro, underwent chest radiography, regardless of their serological status, for the presence of *D. immitis* antigen, and none of them used preventive measures. Eight dogs tested positive for the antigens on immunochromatographic tests (8/15, 53.3%). When the radiographs of the two groups of dogs were blindly compared, the increase in the caudal pulmonary arteries showed a strong positive correlation (0.732) with infection using the Spearman correlation test. These results suggest that heartworm infection, even in the absence of clinical signs, elicits caudal lobar pulmonary artery enlargement that can be detected on radiography.

## Resumo

A dirofilariose canina é responsável por gerar importantes alterações pulmonares, mesmo em pacientes assintomáticos. Este estudo tem como objetivo comparar análises radiográficas do tórax de pacientes naturalmente infectados por *Dirofilaria immitis* e de pacientes negativos para a infecção. Quinze animais atendidos em uma clínica veterinária privada no Município de Maricá, no estado do Rio de Janeiro, foram submetidos a radiografia de tórax, independentemente de seu estado sorológico para presença de antígeno de *D. immitis*, onde nenhum deles fazia uso de preventivo. Dos 15 animais radiografados, 53,3% (8/15) eram positivos para antígeno de *D. immitis*. Quando avaliadas as radiografias dos animais negativos com animais positivos, o aumento das artérias pulmonares caudais apresentou correlação forte positiva (0,732) com a infecção pelo teste de correlação de Spearman. Os resultados sugeriram que a infecção por dirofilariose, mesmo na ausência de sinais clínicos, provoca aumento da artéria pulmonar lobar caudal que pode ser detectado em radiografias.

## Introduction

Canine heartworm infection (HTW) is a cardiopulmonary parasitic disease caused by the nematode *Dirofilaria immitis* (Leidy, 1856). The parasite is vectored by mosquitoes of the genera Aedes, Anopheles, and Culex. It has assumed global significance, particularly in endemic areas with favorable climatic conditions for mosquito proliferation. Adult worms primarily inhabit the pulmonary arteries and the right side of the heart, causing substantial structural and functional damage to the cardiopulmonary system (Bowman & Atkins, 2009). In highly endemic regions, the prevalence of HTW can reach alarming levels (Scavo et al., 2022). Labarthe et al. (2014) documented high rates of canine heartworm infection in multiple regions of Brazil, emphasizing the importance of constant surveillance and preventive measures.

HTW elicits significant pulmonary lesions even in asymptomatic cases, and chest radiography is an important tool for evaluating the lungs of infected cases (Falcón-Cordón et al., 2024). Radiographic abnormalities, such as enlargement of the caudal pulmonary arteries, pulmonary trunk, and abnormal pulmonary patterns, are frequently observed in HTW-infected dogs and have been correlated with disease severity (Maerz, 2020; Ferreira et al., 2024). However, these findings are not exclusive to HTW and may indicate several other respiratory diseases (Cummings & Wylie, 2019; Sakarin et al., 2024).

In addition to evaluating the pulmonary pattern, thoracic radiography has been shown to be a valuable diagnostic tool in monitoring disease progression and treatment outcomes. In a recent study, Falcón-Cordón et al. (2024) demonstrated significant radiographic improvements in pulmonary lesions following adulticide therapy, highlighting the utility of imaging for both diagnostic and therapeutic evaluations. The present study aimed to compare the chest X-rays of *D. immitis* naturally infected dogs with those of *D. immitis* infection-free dogs living in the same endemic area to identify radiographic markers that could be related to *D. immitis* infections.

## Material and methods

Fifteen dogs treated in May 2024 at a veterinary clinic located in Maricá, a highly endemic area in the state of Rio de Janeiro, underwent chest radiography regardless of their *D. immitis* antigen status and presented no respiratory clinical signs. Infection by *D. immitis* was confirmed by the Uranotest Dirofilaria immunochromatographic test. All animals were evaluated in the ventrodorsal, left lateral, and right lateral positions. The images obtained using the digital radiography equipment (DR Tech and SiUI transmitter) were analyzed by the same trained radiologist and veterinarian in a blinded manner. The cardiac silhouette, pulmonary trunk, caudal lobar pulmonary artery, cranial lobar pulmonary artery, caudal vena cava, and pulmonary patterns were evaluated. The normality of the data obtained was assessed using Shapiro-Wilk tests, with a non-normal distribution (p > 0.05 for all parameters). The assumption variance homogeneity was assessed using Levene’s test. Student’s t-test was performed for independent samples to determine differences in radiographic variables differed between cases positive and negative for *D. immitis* infection. Bootstrapping procedures (1000 re-samplings; 95% CI) were performed to obtain greater reliability of the results, correct deviations from the normality of the sample distribution, and differences between group sizes, with a 95% confidence interval for the differences between the means (Haukoos & Lewis, 2005).

## Results

Of the 15 dogs, 53.3% (8/15) tested positive for *D. immitis* antigen. Of the eight antigen-positive animals, 87.5% (7/8) were female and 12.5% were male (1/8). Most dogs were of a mixed breed (87.5%), and only one purebred dog, a bulldog, was diagnosed with heartworm. The antigen-positive dogs were aged from 5 to 9 years, presenting an average of 7 ± 1.8 years and weighed from 12 to 30 kg, with an average of 21 ± 6.4 kg. Of the seven antigen-negative dogs, 71% were female (5/7) and 29% male (2/7), with an average age of 5.7 ± 3.9 years and an average weight of 18.5 ± 4.1 kg.

The increase in the caudal pulmonary arteries showed a strong positive correlation (0.732) with infection (Table 1), according to Spearman’s correlation test. HTW-infected dogs showed a greater increase in the caudal lobar pulmonary artery than dogs without HTW infection presenting with normal arteries (t(13) = 3.536, p = 0.004) with a significant difference between the two groups (Cohen’s d = 1.8896). Cases negative for *D. immitis* infection presented with radiographic findings such as the presence of bronchial and interstitial patterns and other nonspecific radiographic findings. Animals in the positive group presented with radiographic findings that may indicate lesions caused by the parasite, such as right atrioventricular enlargement and enlargement of the caudal lobar pulmonary artery, the latter being the finding with the greatest correlation with infection.

**Table 1:** Division of the frequency of radiographic parameters observed according to the group of animals.

**Group**	**RAVE**	**P.T. increase**	**CdPA increase**	**CrPA increase**	**CVC increase**	**Bronchial pattern**	**Interstitial pattern**	**Alveolar pattern**	**Micronodular pattern**
Positive group (n = 8)	100% (8/8)	50% (4/8)	87.5% (7/8)	Absent (0/8)	37.5% (3/8)	100% (8/8)	100% (8/8)	12.5% (1/8)	12.5% (1/8)
Negative group (n = 7)	85% (6/7)	14% (1/7)	Absent (0/7)	Absent (0/7)	57% (4/7)	100% (7/7)	100% (7/7)	Absent (0/7)	Absent (0/7)

RAVE, right atrioventricular enlargement; P.T., pulmonary trunk; CdPA, caudal pulmonary artery; CrPA, cranial pulmonary artery; CVC, caudal vena cava.

## Discussion

The radiographic findings of the evaluated dogs highlight the importance of chest radiographs as a follow-up tool in *D. immitis* endemic areas or may include heartworm as an unexpected diagnostic possibility when present in dogs from non-endemic areas. Abnormalities, such as a pulmonary trunk or enlargement of the caudal lobar pulmonary arteries, may be indicative of *D. immitis* infection, even in dogs with no clinical signs, as suggested previously (Maerz, 2020; Cummings & Wylie, 2019; DeFrancesco, 2009). Additionally, the right atrioventricular enlargement observed in the antigen-positive group is consistent with cardiac remodeling caused by the chronic burden of heartworms, as previously described in studies of cardiopulmonary alterations associated with *D. immitis* (Ferreira et al., 2024). However, these findings are not exclusive to heartworm disease and may also be associated with other conditions, such as secondary pulmonary hypertension, interstitial lung disease, thoracic neoplasms, and bronchial obstructions (Sakarin et al., 2024). Thus, a definitive diagnosis of heartworm infection should always be confirmed using specific tests, such as the antigen test and microfilaria detection, in conjunction with clinical evaluation (Cummings & Wylie, 2019; Bowman & Atkins, 2009). Nevertheless, a complete diagnostic workout must be conducted once chest X-ray findings, even in *D. immitis* antigen-positive dogs, suggest that they may be more than one cause.

The absence of clinical respiratory signs in infected dogs suggests that significant pulmonary lesions may occur subclinically, misleading the differential diagnosis. This underscores the importance of incorporating regular radiographic examinations as part of preventive management strategies in endemic areas, even in asymptomatic animals. Therefore, the strong correlation between the caudal lobar pulmonary artery increase and *D. immitis* infection is indicative of heartworm infection, even in areas where heartworm infections are uncommon.

This study has some limitations. Despite the observed correlations, the small sample size limited the generalizability of the results, highlighting the need for studies with larger populations to validate these findings. Additionally, although statistical techniques such as bootstrapping help mitigate issues associated with small sample sizes, they do not completelyeliminate bias (Haukoos & Lewis, 2005). Future studies should include longitudinal analyses and follow-ups of dogs with varying degrees of infection to better understand the progression of radiographic alterations and their relationship with *D. immitis* infection.

## Conclusion

*D. immitis* infection in dogs, even in the absence of clinical signs, can be suspected based on specific X-ray images of the caudal lobar pulmonary artery. The findings presented in this study support the use of chest radiography as a complementary tool for the early diagnosis of pulmonary complications.
